# Changes in specialized blood vessels in lymph nodes and their role in cancer metastasis

**DOI:** 10.1186/1479-5876-10-206

**Published:** 2012-10-04

**Authors:** Ser Yee Lee, Chao-Nan Qian, Aik Seng Ooi, Peiyi Chen, Hui Min Bernice Wong, Swe Swe Myint, Jing Chii Wong, Siok Gek Jacqueline Hwang, Khee Chee Soo

**Affiliations:** 1 Department of General Surgery, Singapore General Hospital, Singapore, Singapore; 2 National Cancer Centre Singapore – Van Andel Research Institute, Translational Research Laboratory, Division of Medical Sciences, National Cancer Centre, Singapore, Singapore; 3 Department of Surgical Oncology, National Cancer Centre, Singapore, Singapore; 4 Laboratory of Cancer and Developmental Cell Biology, Van Andel Research Institute, Grand Rapids, Michigan, USA; 5 Department of Nasopharyngeal Carcinoma, Sun Yat-sen University Cancer Center, Guangzhou, Guangdong, People's Republic of China; 6 Center for Cancer Genomics and Computational Biology, Van Andel Research Institute, Grand Rapids, Michigan, USA; 7 Department of Statistics & Applied Probability, Faculty of Science, National University of Singapore, Singapore, Singapore; 8 Department of Pathology, Singapore General Hospital, Singapore, Singapore

**Keywords:** High endothelial venules, Cancer metastasis, Angiogenesis, Lymph nodes

## Abstract

**Background:**

High endothelial venules (HEV) have been recognized to play a role in metastasis by its changes seen in the cancer microenvironment of lymph nodes (LN) and solid cancers. Squamous cell carcinoma (SCC) of the tongue is a prevalent tumor of the head and neck region with high propensity for LN metastasis. The extent of LN metastasis is the most reliable adverse prognostic factor. Primary tumors can induce vasculature reorganization within sentinel LN before the arrival of tumor cells and HEV represents these remodelled vessels. This study aims to evaluate the cancer induced vascular changes in regional lymph nodes (LN) of patients by studying the morphological and functional alterations of HEV and its correlation with clinical outcome and pathological features.

**Methods:**

This study was based on 65 patients with SCC tongue who underwent primary surgical treatment including neck dissection. The patients were categorized into 2 groups based on the presence of malignancy in their cervical lymph nodes. A review of the patients' pathological and clinical data was performed from a prospective database. Immunohistochemical staining of the tissue blocks for HEV and high-power-field image analysis were performed and analyzed with correlation to the patients' clinical and pathological features.

**Results:**

The total number of HEV was found to be significantly associated to disease-free interval. There was a similar association comparing the HEV parameters to overall survival. The density of abnormal HEV was significantly higher in patients with established metastases in their lymph nodes and HEV was shown to be a better prognosis factor than conventional tumor staging. The HEV morphological metamorphosis demonstrates a spectrum that correlates well with disease progression and clinical outcome.

**Conclusions:**

The results suggest that the HEV displays a spectrum of morphological changes in the presence of cancer and LN metastasis, and that HEV is possibly involved in the process of cancer metastasis. We revealed the relationship of HEV and their metamorphosis in pre-metastatic and metastatic environments in regional lymph nodes of tongue cancer patients in relation to clinical outcomes. The significant observation of modified dilated HEV containing red blood cells in lymph nodal basin of a cancer suggests the shifting of its function from one primarily of immune response to that of a blood carrying vessel. It also demonstrated potential prognostic value. More studies are needed to elucidate its potential role in cancer immunotherapy and as a potential novel therapeutic approach to preventing metastasis by manipulating the remodelling processes of HEV.

## Background

Cancer research has focused significantly on the pathogenesis of metastasis as the presence of metastases often translate to a poor prognosis with relatively few effective therapeutic measures.

Oropharyngeal carcinoma is ranked among the top ten most common cancer diagnosed in men in the United States [1]. Of all the carcinomas of the head and neck, tongue is the most prevalent site. The 5-year survival rate for oral cancer has not improved significantly over the past several decades and remains at 50–55%, despite current advances in surgery and radiation therapy [2,3]. This is primarily because mortality results from metastatic disease and local recurrence.

The first draining lymph node was coined the “sentinel node” 3 decades ago by Cabanas, who defined the concept of the sentinel node being the doorway to the regional node basin [4]. Sentinel lymph node (SLN) metastasis is the preliminary process in the spread of cancer in many malignancies. The sentinel lymph nodes undergo morphological and functional changes induced by the primary tumor. Some of these changes are brought into effect by vasculature and lymph channel reorganizations before the arrival of cancer cells and the key blood vessels in such lymph nodes (LN) that are remodeled are identified as high endothelial venules (HEV) [5]. Tumor-reactive lymphadenopathy in SLN has been observed for decades, but alterations of the lymphatic channels and vasculature in these nodes before the arrival of metastatic tumor cells remain poorly characterised [6,7].

### High endothelial venules and its role in cancer metastasis

There is emerging data elucidating the potential role of specialized blood vessels called HEV in cancer, either as a prognostic factor or as a potential modulator or target in tumor immunotherapy [5,6,8-10]. High endothelial venules are specialized post-capillary venules found in lymphoid tissues, located mainly in the T-call zones such as the para-cortical areas of LN. They are morphologically and functionally distinct from ordinary venules and after the vascular endothelial cells (EC) of the blood–brain barrier, the EC of HEV are the next most well characterized. Each HEV has a prominent peri-vascular sheath; a thick basal lamina and the layer of ECs are tall and plump in appearance [9,11]. High endothelial venules were first characterized and studied extensively in the field of immunology. The evidence suggests that HEV has a central role in lymphocyte trafficking to LN, allowing the entrance of native L-selectin ^high^ cells in to the LN parenchyma and this is largely mediated by chemokines produced in and around HEV such as the peripheral node addressins (PNAds). There is a synchrony between HEV and lymphatic vessels as seen in immunization studies, which reveal that the lymphoid tissue microenvironment is crucial for the maintenance of HEV characteristics during homeostasis and the close association in time, function and space between lymphatics and HEV in the remodeling process after immunization indicate that the two systems are closely related and engage in cross-talk through various factors [9,12-14]. The LN undergo remodeling with changes in complex kinetics of lymph flow, cell content flow, blood flow, HEV gene expression as well as morphologically [12]. Recently, with animal models, it was shown that before the arrival of metastasis in the SLN, there are reorganizations of vasculature and lymphatic channels resulting in the SLN becoming a functional blood vessel enriched organ [5]. These prominent blood vessels are identified as remodeled HEV. The role of HEV in immune function and in a cancer metastasis microenvironment has some differences. In inflammatory conditions, the chief role of HEV in the traffic control of lymphocytes is evident from the presence of lymphocytes in the dilated lymphatic sinuses [9,13]; whereas in tumor-reactive lymphadenopathy, there are few cells, suggesting a different process. There are also studies in murine models to suggest that the movement of tumor cells to LN resembles the normal migration of dendritic cells during immune stimulation, resulting in the term “Tumor cell trafficking” [9,15]. This observation, coupled with the knowledge of the intimate relationship between HEV and lymphatic vessels in LN leads to our proposed hypothesis that HEV might provide the shortcut or a bypass route connecting the vascular and lymphatic system at the level of the SLN. This shortcut route is a speculative hypothesis and requires further research. This study aims to provide some preliminary observations in the changes of HEV morphology in a cancer environment and to provide some consistent clinical and morphological data to support our hypothesis [10]. This is in contrast to the traditional Halstedian philosophy that an enbloc resection of the primary cancer and its regional lymphatic basin will achieve cure as its assumption is that tumor cells follow a stepwise pathway, from the primary tumor to the regional lymph nodes and to the next echelon and then to the systematic circulation through distal lymphaticovenous connections such as the thoracic duct. While this orderly fashion is true in the majority of the time, this stepwise progression is not strictly followed as shown in clinical follow-up studies, where up to 20% of women with node-negative breast cancer go on to develop distant metastases [16,17]. This is consistent with the analysis of sentinel lymphadenectomies suggesting that 20% of systemic metastases are derived from cancer cells that bypass this orderly lymphatic route [18]. The HEV providing the vehicle of this shortcut route for cancer cells might account for this observation in this subset of these patients [6].

In addition to this hypothetical role of providing the physical shortcut route in the LN, evident from the presence of significant abundance of red blood cells within the HEV and significant increase size of its lumen in the presence of a tumor, hints that HEV presumably functions like a blood vessel in anticipation to supply the needs for an accelerating growth of a soon-to-arrive tumor deposit [5,10].

### Objectives of study

We aim to evaluate the tumor-induced vascularization in regional LN of the patients with tongue cancer, specifically looking at HEV. We also seek to confirm the morphological and functional alterations of HEV in the regional lymph nodes of the patients with carcinomas of the tongue and correlate these findings with clinical outcome. The key blood vessels involved in LN in a cancer setting are HEV, with its function shifting from immune response to one as a blood flow carrier. We hypothesize that the transformation of HEV in the presence of cancer is a spectrum and these morphological features of altered HEV correlate with clinical outcome of patients, establishing its pivotal role in the pathogenesis of metastasis [6]. This association will serve HEV as a novel prognostic marker and a potential candidate for therapeutic targeting.

## Methods

Approval was obtained from the Institutional Review Boards of both institutions involved in the study (2007/464/B). The study population consisted of 175 consecutive patients with SCC of the head and neck who had received primary surgical treatment at the Department of General Surgery, Singapore General Hospital and the Department of Surgical Oncology, National Cancer Center, Singapore, from January 2001 through to December 2005. The patients’ pathological and clinical data including follow-up information was reviewed from a prospective database. The inclusion criteria included all surgically treated patients with histologically proven SCC tongue who underwent a neck dissection, with a minimum of 2-year follow up period. Patients with a second primary cancer and patients who did not have a neck dissection as part of their primary treament were excluded. There were a total 65 patients that met the study inclusion criteria. There were 35 patients in the group with primary SCC tongue without pathologically proven lymph node metastases in their neck dissection specimen who were designated as “cases”, and 30 patients in the group with primary SCC tongue with pathologically proven lymph node metastases in the neck dissection specimens, who were designated as “controls”. All patients included had radical excision of the primary tongue lesion and a neck dissection according to departmental protocol. In both groups, all patients had radical enbloc excision of the primary tongue lesion with or without resection of adjacent structures with either a unilateral or a bilateral, supraomohyoid neck dissection or a modified radical neck dissection. Tumours were classified according to the AJCC TNM Staging Classification, based on a pre-operative clinical evaluation and this determined the type of neck dissection performed by the primary surgeon [19]. All cases were discussed at a multi-discplinary head and neck tumor board. Intra-operative frozen sections were performed for margins of the tongue lesion and further resection of the tongue was performed in event that the surgical margins were involved. Upon discharge from the hospital, they were followed up at regular intervals of monthly, 3-monthly, 6-monthly and yearly at a progessive rate. Inpatient and outpatient clincal data, pathological, radiological and operative records were retrieved from a central medical records office and from the Department of Pathology, Singapore General Hospital. Mortality data was obtained and confirmed from the hospital central medical records office as well as the Singapore Registry of Births & Deaths.

### Immunohistochemistry

A histological review and analysis of the primary SCC tongue lesion and their respective lymph node tissues in the neck dissection specimen from the 65 cases of tongue cancer patients was conducted. The lymph node tissues in each case were separated into levels according to their anatomical locations. The tumor and the neck dissection paraffin tissue blocks from the selected patients were retrieved from the Department of Pathology, Singapore General Hospital. An independent head and neck pathologist reviewed the histological specimens and selected the 3 largest lymph node in both groups for staining of the HEV. The largest 3 LN were chosen as the size of a lymph node correlates with the degree of tumor lymphadenopathy and in the cases of the non-metastatic neck, the degree of reactive lymphadenopathy. Optimisation of the immunohistochemical staining protocol and subsequent staining of the HEV using the purified rat anti-mouse MECA-79 (PNAd) antibody (BD PharMingen, CA, U.S.A), which is specific for HEV, was performed [5,20].

### Computer assisted image analysis

The digital capture of the HEV images and the quantitative image analyses were performed using the slide scanner (ScanScope, Aperio T3; ScanScope Console v. 9.0. Aperio Technologies, Vista, CA U.S.A) utilising an illuminator(Fiber-Lite DC-950, Dolan Jenner, Boxborough, MA, U.S.A). We analyzed snapshots of the largest 3 lymph nodes under 10x magnification. The total number of HEV, abnormal HEV (defined as having a lumen area of 80 square microns) and the HEV with presence of red blood cells were counted with the aid of imaging software (Image J programme with cell counter plug-in, NIH, Bethesda, MD U.S.A) [21]. The lumen size of 80 square microns was selected as it is the minimum luminal cross-sectional area for functional vessels, corresponding to the minimum caliber of physiologic venules [5]. An average value for each of these parameters was calculated for each LN. These 3 values were correlated to the size of its respective LN and the patient’s clinical outcomes, namely the overall survival, disease-free interval and recurrence rate.

### Definitions of the HEV’s parameters and ratios

**Figure 1:**
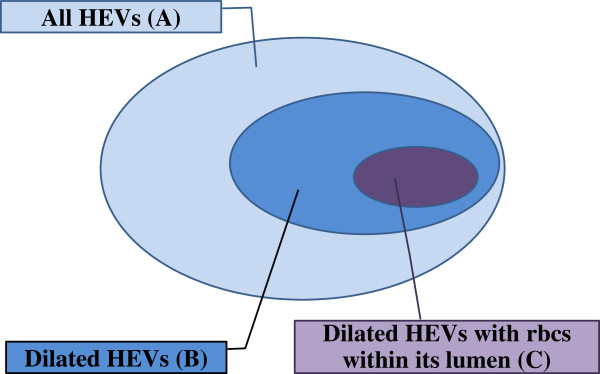
**The different High endothelial venules parameters.** Venn diagram illustrating the relationship between the different HEV parameters (**A, B, C**).

This is represented by the following alphabets A, B and C (Figure 1).

• number of all HEV : A

• number of dilated HEV (defined as lumen size more than 80 square micron) : B

• number of dilated HEV with red blood cells (RBC) within its lumen : C

In addition to these three parameters, we also analyzed the ratios of these abnormal HEV (B and C) with respect to the total number of HEV (A). This is summarized below as

• Ratio of dilated HEV to the total number of HEV : B/A

• Ratio of dilated HEV with RBC within its lumen to total number of dilated HEV : C/B

• Ratio of dilated HEV with RBC within its lumen with respect to total number HEV : C/A

### Statistical analysis

Correlating the clinical information from our prospective database, we analyzed the 3 parameters (A, B, C) and the 3 ratios (B/A, C/B, C/A) with respect to several clinical parameters, namely Disease-Free Interval (DFI) and Overall Survival (OS) in the 2 groups (Cases vs. Controls) and as a single cohort. We used Cox’s Proportional Hazard Model for analysis. In the secondary analysis, we analyzed the 3 parameters (A, B, C) and the 3 ratios (B/A, C/B, C/A) with tumor characteristics. A non-parametric test, Wilcoxon-rank-sum test, was performed to test the difference on HEV and its parameters as well as 3 ratios between the 2 groups of patients. A non-parametric test, Kruskal-Wallis test, was performed to test the difference on the HEV as well as 3 ratios among different stages. All p-values of < 0.05 were considered statistically significant.

## Results

The association of HEV and clinical outcome was studied by initially analyzing the OS and DFI of the 2 groups prior to individually analyzing the various HEV parameters (A, B, C, B/A, C/B, C/A) against OS and DFI. The details of the preliminary results comparing HEV parameters and patient outcomes have been previously published in an earlier paper [20]. In the secondary analysis, we further analyzed the tumor pathological characteristics (i.e. primary tumor volume, the stage of the disease and grade of the tumor) against clinical outcome (OS and DFI), to determine if these conventional tumor factors were prognostic in nature. We further investigated the role of HEV and its prognostic value by comparing and analyzing the HEV parameters with respect to the tumor volume, grade and stage. These analyses were repeated without controlling for the group factor (i.e. taking the sample population as a single cohort). Finally, we investigated the HEV parameters in each group to determine if there was a difference in the HEV values in the presence or absence of metastasis in the LN.

We analyzed the DFI and OS of the 65 patients with respect to their groups (35 cases versus 30 controls) to estimate the relative risk of DFI and OS associated with the group.

### Overall survival analysis

**Figure 2:**
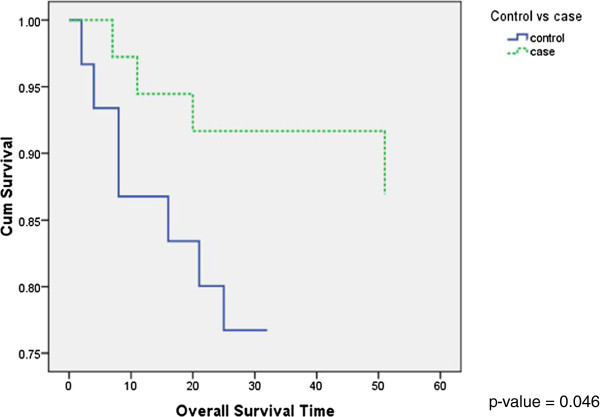
Kaplan Meier overall survival curves for the two groups (Cases vs. Controls).

The relative risk of OS of cases was estimated to be 0.229 times of the risk of control group based on the study sample (95% C.I. 0.048 ~ 1.102). Patients with presence of metastases in their regional cervical LNs had a 4.367 times risk of mortality as compared to patients without metastasis. The Kaplan -Meier curves were significant in terms of overall survival time between controls and cases (p = 0.046) (Figure 2). There was no significance detected found in the total no. of HEV (A); no. of dilated HEV (B); no. of dilated HEV with RBC inside its lumen(C) or any ratios(B/A, C/B, C/A) with respect to the overall survival (Table 1).

**Table 1:** Summary of results

**HEV parameters**	**Clinical data**	**Relative risk**	**p**	**95% Confidence interval**
Total no. of HEV (A)	Overall Survival	1.024	0.471	0.961 ~ 1.091
	Disease Free Interval	1.051	0.022	1.007 ~ 1.097
Total no. of HEV (A) and Disease Free Interval (as a Cohort)	Disease Free Interval	1.051	0.023	1.007 ~ 1.096
Dilated HEV (B)	Overall Survival	1.071	0.476	0.886 ~ 1.295
	Disease Free Interval	1.034	0.594	0.915 ~ 1.169
HEV with RBC within its lumen (C)	Overall Survival	1.116	0.345	0.889 ~ 1.401
	Disease Free Interval	1.044	0.584	0.896 ~ 1.216
Ratio of dilated HEVs to the total no. of HEV(B/A)	Overall Survival	1.078	0.982	0.002 ~ 638.23
	Disease Free Interval	10.10	0.450	0.001 ~ 39.89
Ratio of dilated HEVs with RBC to total no. HEV (C/A)	Overall Survival	3.624	0.737	0.002 ~ 6634.42
	Disease Free Interval	4.67	0.643	0.001 ~ 145.83
Ratio of dilated HEVs with RBC within its lumen to total no. of dilated HEV (C/B)	Overall Survival	17.884	0.171	0.287 ~ 1114.67
	Disease Free Interval	5.458	0.208	0.389 ~ 76.616

### Disease free interval analysis

**Figure 3:**
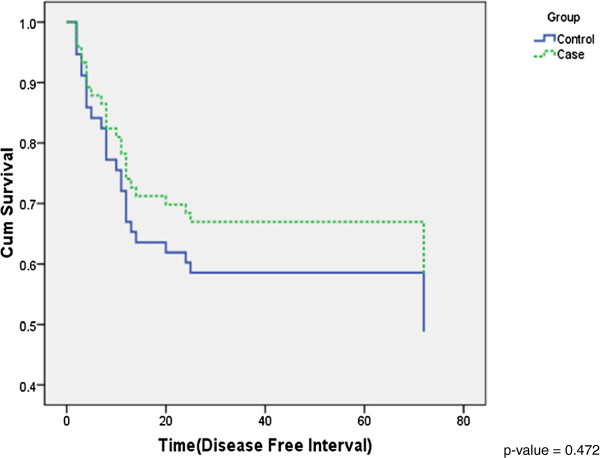
Disease free interval curves for the two groups (Cases vs. Controls).

Similarly, we compared the DFI between the 2 cases and controls. The risk of cases was estimated to be 0.75 times of the risk of control group based on the sample (95% C.I. 0.342 ~ 1.644). This implied that in patients without LN metastases, if recurrences occur, the DFI tend to be longer. However, this did not achieve statistical significance (p = 0.472) (Figure 3).

There was a significant association of number of HEVs (A) to the disease free interval (DFI). This was statistically significant when controlling for the group factor (p-value = 0.022), and when analyzing as a single cohort (p-value = 0.023). However, there was no significance detected found in the other HEV parameters in relation to DFI, namely no. of dilated HEVs (B); no. of dilated HEVs with RBC inside its lumen (C) or any of the HEV ratios(B/A, C/B, C/A) with respect to DFI (Table 1).

### Secondary analysis

Tumor pathological data, namely primary tumor volume, the stage of the disease and grade of the tumor were analyzed against OS and DFI, to determine if these conventional tumor factors were prognostic in nature. We did not detect any prognostic value of tumor volume; stage or grade that reached statistical significance (Table 2). As part of the secondary analysis, we compared the HEV parameters in the 2 groups as well as the HEV parameters to the tumor characteristics (tumor stage and its grade only; tumor volume being a continuous variable was excluded). There was no significance detected in either stage or grade of tumor with regards to the HEV parameters. In the final part, we investigated the various HEV parameters in each groups to if there was a difference in the HEV values in the presence or absence of a metastasis in the LN. The average ratio of dilated HEV with RBC within its lumen to total no. of dilated HEV (C/B) in control groups was statistically significantly higher than that in case group (p = 0.0318) (Table 3).

**Table 2:** Summary of secondary analysis results

**Tumor characteristics**	**Clinical data**	**Relative risk**	**p**
Tumor volume (TV)	Overall Survival	0.985	0.476
	Disease Free Interval	0.990	0.481
Stage (S)	Overall Survival	1.116	0.710
	Disease Free Interval	1.302	0.348
Grade(G)	Overall Survival	0.882	0.815
	Disease Free Interval	1.436	0.308
Since there are no statistical difference noted between the 2 groups, we now consider the 2 groups (Cases and Controls) as a cohort © and repeat the analysis summarized below			
Tumor volume (TVc)	Overall Survival	0.994	0.765
	Disease Free Interval	0.994	0.648
Stage (Sc)	Overall Survival	1.364	0.237
	Disease Free Interval	1.209	0.255
Grade(Gc)	Overall Survival	1.121	0.822
	Disease Free Interval	1.493	0.221

**Table 3:** Comparing high endothelial venules parameters in the 2 patient groups

**HEV parameters**	**Control**	**Case**	**p value**
Mean/standard error	(n = 30)	(n = 35)	p>0.05
Total number of HEV (A)	40.085 / 1.83	41.305 / 1.59	p>0.05
Ratio of dilated HEV to the total number of HEV (B/A)	0.149 / 0.0086	0.166 / 0.0186	p>0.05
Ratio of dilated HEV with RBC within its lumen with respect to total no. HEV (C/A)	0.098 / 0.0078	0.101 / 0.0135	p>0.05
Ratio of dilated HEV with RBC within its lumen to total no. of dilated HEV (C/B)	0.646 / 0.0301	0.582 / 0.0275	p=0.0318

## Discussion

Squamous cell carcinoma of the tongue has one of the fastest rising rate today affecting the young with a high propensity for LN metastases. [22] The presence of metastatic cells in regional LN, along with extracapsular spread are the most important prognostic factors in patients with squamous cell carcinoma (SCC) of the tongue [22-25]. The prognosis is worse compared to other equivalent carcinomas of the head and neck region.

Tumor cell metastasis to regional LN marks one of the initial steps in the cascade of tumor metastasis after tumor cell progression in the primary tumor. The lymphatic metastatic cascade is a series of complex interrelated steps and processes [26]. As the tumor grows and enlarges, cytokines are secreted to promote lymphangiogenesis [27]. As malignant cells invade the extracellular matrix, they enter the lumen of the lymphatics and they move in clusters or as single cells to the first echelon of regional LN, otherwise known as the SLN [28,29]. The structural properties, such as the lack of basement membrane in terminal lymphatics, the presence of the intercellular clefts, the traumatic environment in the blood including shear forces of blood turbulence and tumor antigenicity detection by host immune cells, contribute and account for the observation that in many solid cancers, lymphatic metastasis precedes metastasis via the vascular system [26,30]. The relationship between angiogenesis and lymphangiogenesis has been extensively studied in cancer research [14,26,31,32]. This is pertinent in the biology of LN metastasis where the two systems literally lie side by side. There is extensive evidence showing members of the Vascular endothelial growth factor (VEGF) family, namely VEGF-C, VEGF-D and VEGF-A [33], are not only important regulators of lymph vessel growth in vivo, but may also be involved in the process of promoting lymphatic metastasis, such as VEGF-C [27,34-36]. This is significant because there is evidence that tumor can activate both LN lymphangiogenesis and angiogenesis before metastasis, and this would translate into logical efforts in the field of anti-cancer research specifically targeting pathways of tumor lymphangiogenesis and angiogenesis. There have been encouraging results from therapeutic targeting of the various VEGF member pathways to inhibit lymph node metastasis, reduce lymphatic and vascular density in LN as well as to reduce overall tumor burden in the lymph nodes and distant metastases [34,37,38].

Targeted therapy such as anti-angiogenesis drugs has shown positive results in clinical studies. Bevacizumab (Avastin^TM^), a monoclonal antibody to VEGF-A, was approved by United States Food and Drug Administration (FDA) in 2004 and has proven clinical benefits in treatment of metastatic colorectal cancer when the drug was added to standard chemotherapy and also has positive results in metastatic kidney cancer and advanced lung cancer. There are many ongoing trials including Phase III clinical trials in advanced or metastatic renal cell carcinoma, pancreatic cancer, and ovarian cancer for Avastin^TM^[39]. Other molecular pathways that harbor potential targets which have varying degrees of anti-angiogenesis and anti-lymphangiogenesis properties, are ligands such as hepatocyte growth factor (HGF), receptors like Neuropilin 2, PDGFRα/β and others such as Angiostatin, interferon α/β and platelet-factor 4 [40]. Two other antiangiogenic drugs, Sorafenib (Nexavar™, Bayer) and Sunitinib (Sutent™, Pfizer), have also been approved by the FDA for various aspects of cancer treatment, for example, Sorafenib and Sunitinib have been beneficial in the treatment of metastatic renal-cell cancer and advanced hepatocellular carcinoma [41,42]. They target multiple receptor tyrosine kinases, including VEGF receptors and platelet-derived growth factor (PDGF) receptors [43].

**Figure 4:**
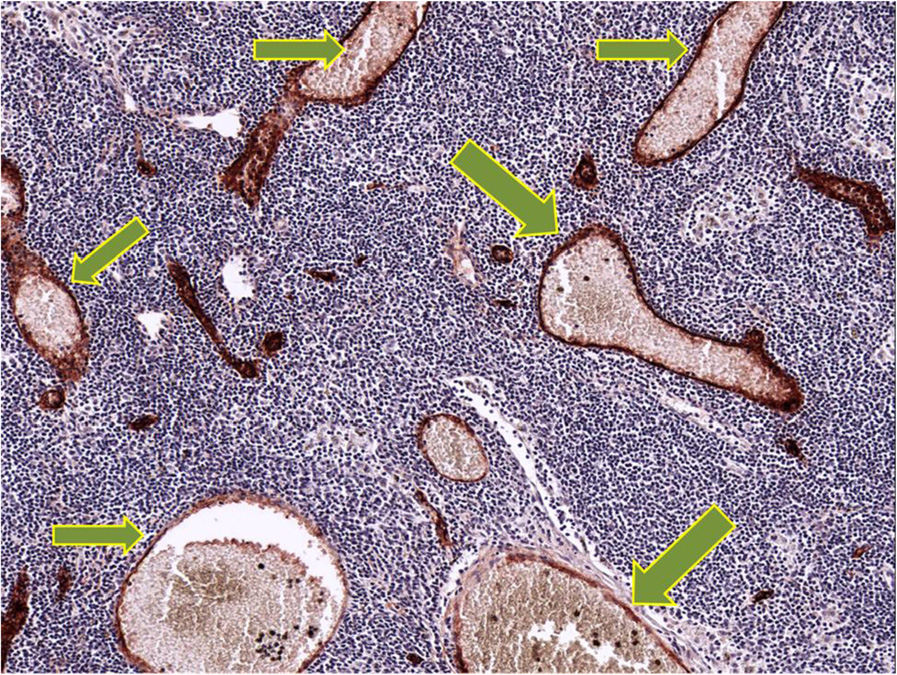
Dilated HEVs with red blood cells in its lumen (high power field).

In our series, we detected a statistical significance when we analyzed the OS but not in DFI in the patients with or without LN metastasis (cases vs. controls) (Figures 2 and 3). This may be attributed to our limited sample size. It has been proposed and is currently being validated, that SLN biopsy may play the next stage in the evolution in the neck management and treatment of tongue SCC [44]. Exploration and understanding of this concept coupled with advances and optimistic results in anti-angiogenesis therapy involving VEGF anti-metabolites translates the next logical exploration to be invested in the study of the pathogenesis of lymph node metastasis. It was shown that the lumen of the lymphatic sinuses and blood vessels were dilated in SLN before metastasis [5]. We have demonstrated that lymph nodes are transformed by the primary tongue tumor to become a functional blood vessel–enriched organ before and independent of metastasis, with the changes in the morphology of the HEV to become main blood flow carrier in the lymph node (Figure 4). This process of vascularization in the LN (HEV morphological alterations) appears similar and consistent in human tissues and previous animal models [5,20]. This study presents functional and structural data that the primary tumor can possibly manipulate the microenvironment and biology of lymph nodes to potentially facilitate the proliferation of subsequent tumor deposits leading to established metastases. The morphological alteration of HEV in the presence of a cancer, coupled with the increased proliferation rate of the endothelial cells, results in a functional shift and phenotypic change of HEV from its known function as a lymphocyte recruitment modulator to one as a blood carrier.

In previous studies, the degree of lymphatic dilation in the SLN significantly correlated with the primary tumor weight [5,10]. This observation suggests that the lymphatic fluid from the primary tumor induced the persistent alteration of the lymph channel in SLN. These results are consistent with findings that demonstrate, in contrast to angiogenesis, in which blood flow proceeds only after the vessel develops, lymphangiogenesis can be induced by interstitial fluid channeling [45]. In this aspect, this is analogous to immunology studies showing the alteration of lymph channels in SLN can also occur during an inflammatory reaction, which facilitates the migration of inflammatory cells [46,47] The difference in cancer metastasis biology and inflammatory process starts here. In endotoxin induced experiments and immunology studies, the dilated lymphatic sinuses and vessels in the associated reactive LN were full of lymphocytes. In contrast in tumor-reactive lymphadenopathy, the lymphatics contained minimum or no cellular material, suggesting different roles of the SLN lymphatic channels in different pathologic processes. It has been well characterized that the HEV of lymph nodes play an important role in recruiting lymphocytes for the generation of immune responses [9,11]. By expressing homing receptors on their surface, which blood lymphocytes can recognize as they pass in circulation, HEV provide a unique location where naive lymphocytes can enter the lymph node [47,48].

In our previous study [5], we found that the role of HEV was transformed from a lymphocyte recruiter to become the main blood flow carrier in the SLN prior to metastasis. We have shown in this study that in the regional cervical LN, not only could the morphology of individual HEV change dramatically to increase blood flow, but the proliferation rate of HEV endothelial cells was also increased before metastasis. This transformation of HEV as a lymphocytic carrier in LN to a blood-flow carrier can be seen in the large quantity of red blood cells visible in the HEV even in pre-metastatic regional LN. More significantly, this phenomenon is also more pronounced in the regional LN of the patients with established LN metastasis. This is consistent with the findings of Chung and colleagues demonstrating the increased HEV density in SLN of oral SCC before the arrival of tumor cells, the HEV density in metastatic SLN was also higher than that of non-metastatic SLN [6]. We have shown this in our supplementary data that patients with lymph node metastases have more dilated HEV with RBC in their LN as compared to patients without LN metastasis. The average ratio of dilated HEV with RBC within its lumen to total no. of dilated HEV(C/B) in the control (pN+) group is statistically significantly higher than that in case group (p-value = 0.0318).

In addition, with the benefit of patients’ pathological and follow-up information, we have demonstrated that even with just an increase of 1 HEV in a high-power-field (HPF), regardless of the group, the risk is 1.024 times worse in terms of overall survival. This is consistent with clinical knowledge and natural history of the disease. It is well established that patients without LN metastases in their regional LN (cases in our cohort) have a significantly better prognosis, and; this fact is also reflected in our analysis [22,49-51] (Figure 2). Cases had a 0.428 times the risk of Controls with regards to overall survival if they had the same number of HEV. It is crucial to understand that this risk is exponential in nature. To illustrate the significance of this result, an example will be used: Patient A with tongue SCC has proven LN metastases in the cervical LN. On further immunohistochemical staining, it is found that he has 0 HEV in a random high-power-field. Comparing this to patient B who has the same grade, stage of tumor, degree of LN metastases as patient A but has 100 HEV in a HPF, patient B will have a [ (1.024)^100^ = 10.715 ] 10 times worse prognosis in OS as compared to patient A. We will then consider patient C who has tongue SCC with no LN metastasis. If the HEV amount in a HPF is the same as either patient A or B, patient C’s risk as compared to either patient A or B will be 2.34 times (1/0.428= 2.34) better in terms of overall survival (Table 4). Although this did not reach statistical significance (p=0.471), we believe this to be a factor of a small sample size.

**Table 4:** Differences in OS and DFI relative risk in the 2 groups

**Patient group**	**Number of normal HEV(A) in a HPF**	**OS Relative risk**	**DFI Relative risk**
Control	0	1.00	
	1 (patient A)	1.024	1.051
	100 (patient B)	(1.024)100 = 10.715	(1.051)100 =144.634
Case	0 (patient C)	0.428	0.732
	1	0.428 × 1.024 = 0.435	0.732 × 1.051 = 0.769
	100	0.428 × (1.024)100 = 4.586	0.732 × (1.051)100 = 105.872

**Figure 5:**
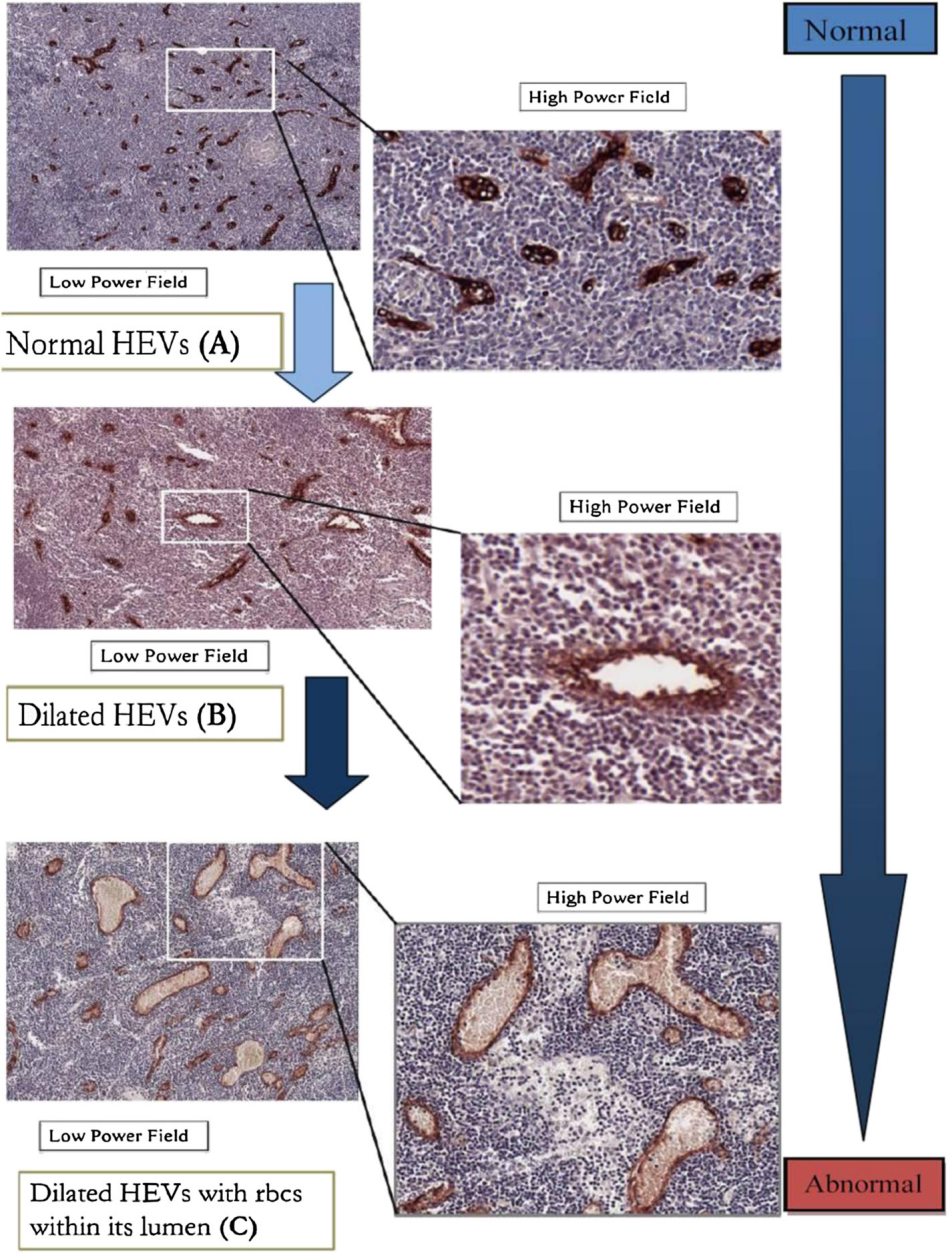
**Metamorphosis of High endothelial venules in a tumor microenvironment.** This process begins with the high endothelial venules increasing in absolute numbers, subsequently each of them becoming more dilated and lastly every one of them will become a function vessel carrying blood.

Assuming that our hypothesis is true, HEV transformation from a normal immunological mediator to a tumor metastasis mediator is reflected by morphological changes from a normal appearing HEV, to a dilated HEV, and then to a dilated HEV containing RBC. We believe that this metamorphosis occurs on a spectrum, and this process begins with the HEV increasing in absolute numbers, then each becoming more dilated before progressing into a functional vessel carrying blood (Figure 5). Hence we analyzed our data looking at the 3 different stages of HEV transformation and the ratio comparing each parameter in a systematic way to demonstrate this spectrum (Figure 1).

We recall that that in the last example we illustrated that a patient’s OS risk is 1.024 times worse than an equivalent patient if there is 1 more HEV in one HPF of his regional LN, the risk is increased to 1.071 if the HEV is a dilated HEV. The risk is even higher at 1.116 times if the HEV is a dilated HEV containing RBC (Table 1) [20]. This trend is also seen when all patients are considered in a single cohort. A patient’s OS risk is worsened by 1.004 times if there is an increase of 1 dilated HEV in a HPF. If that dilated HEV contains RBC, the OS risk is increased to 1.008 times.

In the treatment of tongue carcinomas, DFI is important as it signifies the failure of loco-regional control for which there is little effective therapy. Further surgery e.g. neck dissection to remove loco-regional recurrences is plagued with high morbidity and mortality. This, coupled with poor chemotherapy and radiotherapy response rates in recurrences, often equates to rapid deterioration of the patient’s condition. Disease free interval was also analyzed in the same manner to the three different HEV morphological phenotypes.

We found statistical significance in the relationship between the total number of HEV (A) and DFI when we controlled for the group (i.e. taking in the presence of LN metastasis as a factor). (p=0.022). This significance is preserved when we analyzed all the patients as a cohort (p=0.023) [20]. This means that the quantity of HEV is inversely related to DFI. If you consider the LN status and take it into statistical consideration, if there is one more HEV in a HPF, the DFI is 1.051 times worse than zero HEV. It is again important to note that the relationship of the quantity of HEV with respect to DFI is exponential in nature. For example, a patient (patient B) with 100 more HEV (A) in one HPF has a {(1.051)^100^ =144.634} 144 times shorter DFI as compared to an equivalent patient with only one HEV per HPF (patient A) (Table 4). Excluding the group effect and considering all the patients as a cohort, a patient with 1 more HEV per HPF will have a DFI that is 1.051 times worse than a patient with zero HEV in a HPF (p=0.023).

We looked at the different ratios of abnormal HEV and compared them to several clinic-pathologic parameters namely overall survival, disease–free interval, tumor volume, stage and grade of the tumor. The details are specified in the secondary results (Table 2).

**Figure 6:**
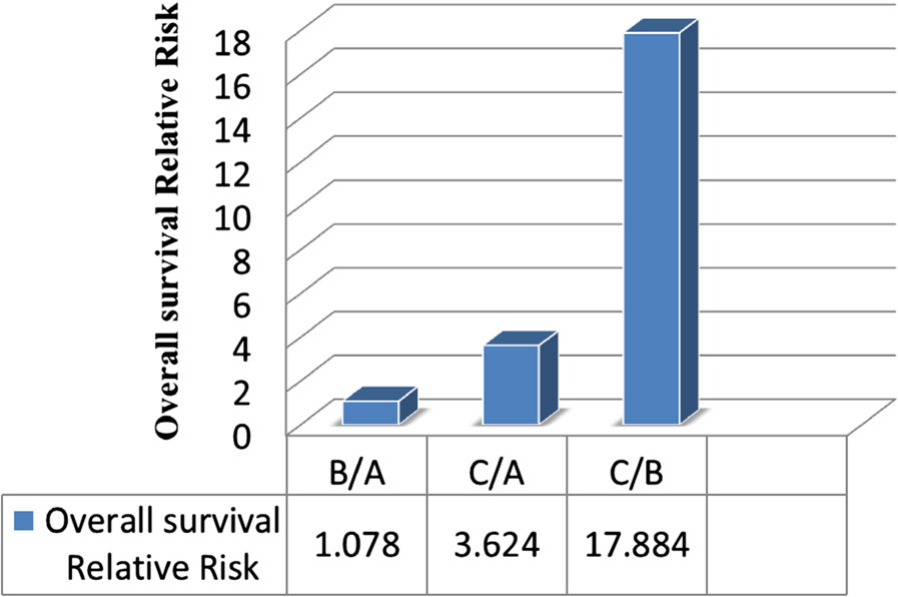
Overall survival relative risk with respect to the different high endothelial venules ratios.

There is a general trend observed. The more advanced the disease, the higher the ratio/percentage of abnormality of HEV. This can be seen in when we analyzed OS and the 2 ratios. The OS relative risk worsens by 1.078 if the ratio B/A (ratio of dilated HEV to the total number of HEV) increased by 1, while the OS relative risk worsens by 3.624 times if the ratio of dilated HEVs with RBC to the total number of HEV increases by 1. Most importantly, if we consider the most abnormal form of HEV (dilated HEV with RBC within its lumen, C) and look at it as a ratio to the total no. of HEV, a patient’s OS relative risk worsens by 17.884 times if this ratio (C/A) increases by a factor of 1. This observation approaches marginal significance. (p = 0.171) (Figure 6).

In this study, we have shown the relationship of HEV and their transformation in a cancerous environment. We also note that in previous studies the HEV morphology did not alter at all in endotoxin-induced lymphadenopathy, implying a selective reaction of HEV in the cancerous condition [5]. Distinct, differentiated gene expression has also been reported when the endothelial cells respond to the changing of their microenvironment [52]. In a previous study, it was shown that the cellular morphology of the tall endothelial cells forming HEV changed dramatically to become flat endothelial cells in the presence of cancer. As a consequence, the HEV was remodelled from a thick-walled, endothelial vessel with a small lumen to a thin walled, large-lumen vessel shifting its function from recruitment of lymphocytes to becoming a blood vessel (Figure 4) [10]. These facts indicate that the blood vessel endothelium has tremendous potential to adapt its environment. The confined lymph and blood channel alterations within the SLN, but not in the next station lymph node, imply that an inducer from the primary tumor is functioning locally with the existence of an active primary cancer. VEGF-A has been found to be an inducer of lymphangiogenesis in SLN [33]. Other studies have shown that serum level of VEGF-A was elevated in patients with late stage NPC [21] VEGF-A is a secreted protein factor that can travel in blood to other lymph nodes, these findings suggest that there may be other inducers involved. It is also of interest to explore the role of HEV after the establishment of a metastatic tumor nest. The enlarged, remodelled HEV could integrate into the metastatic tumor vasculature with further differentiation, characterized by the gradual loss of their specific marker MECA-79 from the tumor margin to the central part of the metastatic tumor nest. [5,10] It has been explained that, compared with primary tumors, the more rapid growth of metastatic lesions in the cervical lymph nodes of NPC patients was due to clonal selection of the cancer cells during metastases, with highly proliferative clones disseminated to the cervical nodes. However, based on our findings, the metastatic tumor vasculature in lymph nodes consists of many large blood vessels derived from normal HEVs, suggesting that the efficiency of nutrition and oxygen supplies could be better for the metastatic tumor cells in the involved lymph node. The enrichment of the blood supply in the lymph node before and after metastasis may favor the growth of newly arriving metastatic cancer cells. Consequently, as evident clinically in the long term follow-up of cancer patients, the involved regional lymph nodes may become manifest first, whereas the primary tumor remain clinically occult for years [53]. Moreover, the high density of functioning blood vessels in lymph nodes may subsequently facilitate the metastasis of cancer cell to distant organs. Conversely, consistent with its role in lymphocyte trafficking, if the HEV density is increased in the tumor themselves as opposed to LN, it has favorable prognostic value, Martinet et al. reported that in 146 breast cancers, the density of tumor HEV was an independent prognostic risk factor for DFI and OS [8]. This further suggests the tumor HEV function as major gateway for lymphocyte infiltration into tumors and represents potential targets for cancer immunotherapy. It will also be important to elucidate if the highly vascularized premetastatic SLN is associated with an increased metastatic potential. Control of lymphatic fluid movement may also be a target to consider in preventing metastases.

Assuming our hypothesis is true with regards to the role of HEV in cancer metastasis, a potential therapeutic approach is to interrupt or block this remodelling process and evaluate if there is an effect on metastasis. This will confirm the HEV’s central role in the pathogenesis of metastasis.

Further studies need to designed and performed to elucidate the pathways of HEV transformation. A control gene for the growth and differentiation of HEV remains to be identified [9]. With regards to this, a recently identified nuclear factor (NF), NF-HEV has potential because it is preferentially expressed by HEV [54]. Dendritic cells, well known for their antigen presentation roles has recently been shown to modulate the phenotype of HEV and control entry of naive lymphocytes to LN, their role in a cancer microenvironment is also an area of potential research [13]. There are recent experiments in mouse models investigating conditional gene targeting of HEV as well [55]. Identification and characterization the molecular pathways and the control genes would have considerable clinical applications. For example, it would enable the design of experiments and studies to induce or retard the formation of HEV in various tissues, including tumors and their LN, improving vaccination strategies against pathogens and cancers. Therefore, there is a wealth of translational applications with regards to targeting HEV as a ligand for cancer investigational therapeutics. It paves the journey in the discovery of many unknown genes and molecules with potential clinical importance. Lastly, more work can be performed to validate our findings in other solid cancers.

### Limitations

Our study did find some preliminary associations in HEV with regards disease pathology and clinical correlations and demonstrated the HEV phenotype changes in cancer; however, it is premature to draw any strong conclusions. Our study has limitations and there are mainly associated with 2 major factors. It is a retrospective study with a limited sample size and its lacks a molecular experimental component.

It is an investigative and proof-of-concept study as the exact role of HEV role in cancer metastasis is still under investigation. The limited sample size of 65 patients is derived over a period of 5 years and it is a subset of a large group selected from the Head and Neck service database in 2 large tertiary institutions. This sample population size was deemed to be sufficient to show a significant difference in their overall survival between the 2 study groups. Nonetheless, in an effort to reduce confounders and errors in the interpretation of our results, a strict selection criteria was applied to obtain a more homogenous group. Due to the small sample size, we believe that some of the associations in our hypothesis did not reach statistical significance. The retrospective nature of the study is no doubt a limitation but as the nature of this study does not involve therapeutics or a comparison of factors, the retrospective nature actually works to our advantage. We are able to collate a large sample study size with confirmatory clinic-pathological data within a short period of time and concentrate our time and effort in the investigative and analytical aspects.

Secondly, one main investigative feature of our study concentrated largely on the histopathological assessment of the patients’ LN status preserved tissue paraffin blocks in storage. There are limitations to using tissue paraffin sections and the available techniques have their limitations. There are deficiencies in the consistency of the quality of the tissue paraffin blocks, some tissue blocks are less well preserved than others, especially the older blocks may not be stored in optimal conditions over time. We use an optimized technique for our immunohistochemistry using anti-MECA-79 as our sole antibody for HEV [5]. Ideally we would like to use at least 2 different HEV markers to confirm our findings in event of specificity and sensitivity inaccuracies in the antibody, however, there is no other commercially available antibody for HEV to date. One assumption in the study is that MECA-79’s specificity and sensitivity is high enough and representative of all HEV in our tissues. Ultimately, analysis at the level of molecular genetics will be the critical factor in confirming the value of immunohistochemical stains in the assessment of biological behavior and prognosis. A considerable problem to be overcome is the marked variation in tissue staining that can be encountered, both in different, patients, neoplasms and in different laboratories. These differences reflect the varied biology of neoplasms, as well as differences in fixation and technique. These variations make comprehensive interpretation of the data and results a worthwhile challenge.

A major limitation in the study is the lack of experimental evidence and results to strengthen our observatory data. The next step in proving our hypothesis is to establish molecular pathway experiments and studies to elucidate the pathways and the molecular mechanisms underlying the phases of HEV’s metamorphosis peri-metastasis. In order to systematically study the role of the modified HEV as a blood vessel and a shortcut mechanism for metastasis, appropriate tracer molecules with real time imaging technology and xenograft experiments studying the mechanism of the lymph node microenvironment changes correlating to the spreading of cancer cells should be pursued. As the objective of this study is to establish the presence of a correlation between HEV and clinico-pathological features in cancer patients, the molecular experiments are not included but are planned for as stated in our future directions.

Finally, ideally the best controls should include neck LN dissection specimens in patients without cancer to optimally illustrate the spectrum of HEV changes from a normal to a pre-metastatic stage and finally to a metastatic one however in reality, it is rare to obtain such specimens and thus not included in the study.

## Conclusion

This study demonstrates morphologic and functional alterations of the HEV to become the main blood flow carrier in the lymph node. The analysis reveals the relationship of HEV and their metamorphosis in pre-metastatic and metastatic environment in regional lymph nodes of tongue cancer patients in close relation to clinical outcomes. Our findings coupled with studies elucidating the basis of lymphangiogesis and angiogenesis within SLN support our hypothesis that HEV play a pivotal role and may be the elusive junction providing the lymph flow shortcut route into the systemic circulation at the level of the SLN. The confirmation of this shortcut and the exploration of the related molecular mechanism of establishing the shortcut will broaden our knowledge about lymph circulation in cancerous conditions and may provide novel therapeutic targets.

## Abbreviations

HEV: High endothelial venules; LN: Lymph nodes; RBC: Red blood cells; SLN: Sentinel lymph node; SCC: Squamous cell carcinoma; OS: Overall survival; DFS: Disease free survival; PNAd: Peripheral node addressin; VEGF: Vascular endothelial growth factor; EC: Endothelial cells; FDA: Food and drug administration; HGF: Hepatocyte growth factor; PDGF: Platelet-derived growth factor; NPC: Nasopharyngeal carcinomas; HPF: High-power-field; NF-HEV: Nuclear factor - High endothelial venules.

## Competing interests

The authors declare that they have no competing interests, financial or non-financial.

## Authors’ contribution

LSY, QCN, SKC conceived the concept, study design and designed the experiments. LSY, OAS, WJC, WHM, MSS collected the data and performed the experiments. SKH, LSY performed the histopathology review: LSY, CP, QCN analyzed the data. LSY, QCN wrote the manuscript. QCN, SKC supervised and critically reviewed the manuscript: All authors have contributed significantly, read and approved the final manuscript.
